# Hereditary bleeding disorder, factor ix deficiency in females: a case series

**DOI:** 10.4076/1757-1626-2-8940

**Published:** 2009-09-18

**Authors:** Kusum L Mishra

**Affiliations:** 1 Department of Pathology, Coagulation Lab, C S M Medical University, Lucknow 226 003, Uttar Pradesh, India

## Abstract

**Introduction:**

Hemophilia is uncommon in females and there is little knowledge about the clinical manifestation.

**Case presentation:**

We report here an unusual case of three hemophilic females diagnosed as factor IX deficient. Normal reports of ultrasonography (USG) and relevant endocrine investigations conducted in two adult females ruled out any usual gynecological and endocrinal causes of bleeding. Complete coagulation profiles were conducted and diagnosed these female bleeders to be hemophiliacs suffering from factor IX deficiency.

**Conclusion:**

Females presenting with menorrhagia and bleeding from other sites without any discernable cause require proper evaluation for congenital coagulation disorders. In the present case series, females are diagnosed factor IX deficient (Hemophilia B).

## Introduction

Menorrhagia is a frequent clinical manifestation of several rare congenital disorders of blood coagulation and platelets [1]. Women with heavy menstruation might not seek medical advice due to unawareness hence the objective assessment of menstrual loss is must-using Pictorial Blood Assessment Chart (PBAC). Usually, 40% of women with losses greater than 80 ml may consider their period as moderate or scanty, conversely, 60% of women with losses <20 ml may consider their period as "heavy". Universally, menstrual blood losses >80 ml is considered as heavy bleeding, therefore, PBAC with semi quantitative assessment of blood losses method adopted to assess the real and false menorrhagia. Awareness in clinicians is also required as menorrhagia is a common problem in women with inherited bleeding disorder and that can be presenting symptom. Hemophilia B is X linked bleeding disorder caused by partial or complete deficiency or dysfunction of factor IX resulting from a variety of defects in the FIX gene [2]. Hemophilia B is reported to affect 1 in 1,00,000 male births in the U.S. and 1 in 30,000 males birth in UK [3]. while, prevalence and morbidity in females with menorrhagia in inherited bleeding disorders have been poorly investigated. Currently 40% to 60% menstruating women complain of menorrhagia with a significant impact on their quality of life, estimated to be of type 1 or 2 and more than 60% of women with type 3 vonWillebrand Disease [4]. In cases, where there is no family history of the disorder the condition is the result of a spontaneous gene mutation. Factor IX deficiency is 4-6 times less prevalent than factor VIII (FVIII) deficiency [3]. Generally the presenting symptoms by patients suffering from hemophilia B is bruising, spontaneous bleeding, bleeding into joints associated pain with swelling and hemorrhage in gastrointestinal tract and urinary tract leading to blood in the urine or stool, prolonged bleeding after injury, tooth extraction, and surgery. Biochemical and Pathological examination in present study done for jaundice, other signs of liver failure (e.g. cirrhosis from viral infection), and of opportunistic infections in patients who are HIV seroconverted to rule out other cause of menorrhagia. No secondary acquired cause of bleeding found. The screening and diagnostic test based upon abnormal Activated Partial Thromboplastin Time, its correction study and factor assay with deficient plasma/serum and severe factor IX deficient plasma used for factor level assays [5]. Normal prothrombin time (PT) and abnormal activated partial thromboplastin time (APTT) ruled out the other vitamin K dependent disorder and common pathway defect.

## Case presentation

### Case report 1

An Aryan Hindu 33-year's-old Indian married woman, complained menorrhagia since last eight years. She could not assess herself to be heavy bleeder therefore; she went to her family gynecologist to have her view regarding the inconvenience she was facing from the last eight years. The ultrasonography (USG) and relevant endocrine investigations revealed no hemorrhagic ovarian cysts or endometriosis, which may also be the common causes of bleeding in women. She has neither the family history of bleeding nor she herself bleed from any other site. Reference sent to our lab for complete coagulation profile. Pictorial blood assessment chart (PBAC) with semi quantitative assessment of blood losses method adopted to assess the real and false menorrhagia. Monthly assessment revealed flooding of blood for more than 80 ml. Screening test resulted in normal prothrombin time (PT), platelet counts (P/C) and function, fibrinogen and thrombin time (TT) with prolonged activated partial thromboplastin time (APTT). This indicated intrinsic pathway defect and ruled out deficiency of vitamin K and Factor V. Mixing study (diagnostic test) showed deficiency of factor IX and the factor assay revealed 42% factor IX activity level (mild deficiency) (Table 1).

**Table 1:** 

Case No.	Screening test			Diagnostic test correction study			Factor IX assay	Fibrinogen* (normal range 200-400 mg/dl)
						
				Mix of Factor IX def. plasma and test plasma (1.1)	Mix of Factor VIII def. plasma and test plasma (1.1)	Mix of Factor IX def. plasma and test plasma (1.1)		
			
	PT (sec)	APTT (sec)	TT (sec)	APTT (sec)	APTT (sec)	APTT (sec)		
Case 1	C:14	C:24	C:10	26	28	68	42%	288 mg/dl
	T:14	T:52	T:11					
Case 2	C:14	C:28	C:10	28	28	82	37%	310 mg/dl
	T:14	T:68	T:11					
Case 3	C:14	C:24	C:10	25	30	140	26%	325 mg/dl
	T:14	T:85	T:12					

Remarks		Raised APTT indicates intrinsic pathway defect			Correction of APTT with normal plasma and FVIII def. plasma and no correction with FIX def. plasma indicates absence of inhibitor & def. of factor IX.			

Abbreviations: APTT, activated partial thromboplastin time; def, deficient/deficiency; Mix, mixture; PT, prothrombin time; TT, thrombin time. The method used for PT, APTT, TT, and fibrinogen was manual technique using commercial reagent (from Diagnostic Stago) as described by Pitney & Brozovic [5].

* The method used for factor IX assay, was manual technique using commercial factor VIII and IX deficient plasma (from Diagnostic Stago) and as described by Pitney & Brozovic [5] (Semi quantitative assay).

### Case report 2

An Arabian origin Muslim young unmarried Indian, 18-year's-old girl gave the history of prolonged gum bleeding, epistaxis along with the history of menorrhagia since menarche. She was not suffering from any gynecological problem. Nasal examination by ENT surgeon and gum bleeding examined by dental specialist disclosed no primary cause of bleeding from the effective site. She was born of consanguineous marriage, but having no family history of bleeding. Monthly assessment using PBAC showed bleeding more than 90 ml. Patient approached us for complete coagulation profile. Screening test revealed only abnormal APTT indicative of intrinsic pathway defect. Mixing study resulted in deficiency of factor IX. Factor assay revealed 37% factor IX activity level (mild deficiency) (Table 1).

### Case report 3

An Aryan Hindu infant 3-year's-old Indian girl presented with off and on petachae and echymotic patches all over body, followed by epistaxis since one year. Nasal examination by ENT surgeon disclosed no primary cause of bleeding from the effective site. There was no family history of bleeding. Then the reference reached coagulation lab for analyzing coagulation disorder. Screening test as above revealed intrinsic pathway defect. Mixing study and factor assay revealed deficiency of factor IX with 26% of factor IX activity level (mild deficiency).

## Discussion

All the three patients had Liver Function Test report normal (already done from biochemistry laboratory) indicating that patient does not have any acquired conditions causing factor IX deficiency. Further, a normal prothrombin test value in screening test ruled out vitamin K deficiency and vitamin K dependent factor deficiency. None of the three patients received any treatment without prior diagnosis. After detection of the deficiency; the factor IX concentrates infused to replace the defective clotting factor. This normalized the uncontrolled bleeding and improved the hemoglobin condition in all the three cases.

Like other parts, haemostatic plugs also characterize homeostasis in the endometrium and more important role it plays during first 2-3 days of menstruation. Studies reported high prevalence of menorrhagia among females with von Willebrand disease [6]. Factor IX, a vitamin K-dependent single-chain glycoprotein, synthesized first by the hepatocyte as a precursor protein (protein in vitamin K absence); then, it undergoes extensive posttranslational modification to become the fully gamma-carboxylated mature zymogens and secreted finally into the blood [2]. The γ-carboxylation of nearly all of the 12 amino-terminal glutamic residues is most important, since this modification is required for calcium ion binding and full enzymatic activity [7]. A 3-fold variation in the activity of factor IX in plasma is normal. Since factor IX is smaller than albumin, it distributes in both the extra vascular and intra vascular compartments [2]. Characterization of a young female with mild hemophilia B is a novel missense mutation (codon 351, GCT (Ala) → CCT (Pro) of the FIX gene [8]. Menorrhagia therefore, a life threatening hidden disorder can be unusually due to severe and unexpected inherited bleeding disorder with a very high mortality rate if unchecked needs precise screening of coagulation disorder. The study indicates the method of analyzing heavy uterine bleeding and emphasize on screening of coagulation disorder.

## Conclusion

Since substantial number of women complains for menorrhagia after menarche irrespective of their age, therefore investigation of such patients for inherited bleeding disorders before planning for any therapy or undergoing invasive procedures: hysteroscopy and hysterectomy that may lead to a high rate of postoperative bleeding is of utmost importance.

## Consent

Written informed consents were obtained from the first and second patient and written informed consent was obtained from the parents for the third patient for publication of these case reports. Copies of the written consent are available for review by the Editor-in-Chief of this journal.

## Competing interests

The author declares that he has no competing interests.
